# Balint group putting on medical trainees in psychiatric internship: first findings and pilot project at Fribourg University, Switzerland

**DOI:** 10.1192/j.eurpsy.2025.916

**Published:** 2025-08-26

**Authors:** I. Gothuey, C. C. Devaud

**Affiliations:** 1 Adult Service, Fribourg Mental Health Network, Marsens; 2 Fribourg University; 3 Forensic Service, Fribourg Mental Health Network, Fribourg, Switzerland

## Abstract

**Introduction:**

Balint groups are recognized for preventing burn-out and ehanced empathy by general practionners. Since few years, they are also identified for enhancing empathy by medical students for difficult patients. With the introduction of a six weeks psychiatric internship during the master medical studies at Fribourg University, a compulsory participation in four Balint Group sessions was implemented for medical trainees during there clinical psychiatric rotation.

**Objectives:**

Hilighting main psychiatric topics concerns by medical trainees during the first and the second years of implementation Hypothesing about imposition participation for medical trainees versus a freely participation.

**Methods:**

An intervision space between group leaders take place to supervising this new design..Each group leader made a presentation about the groupal dynamic and the broached subjets during their session with medical trainees.

These finding were wrintig, discussed beteween and compiled, in order to target a future enquiry’s questions.

**Results:**

The fisrt year, main topics concern psychiatry’s foundations: what to do when a relative or friend is hospitalized? How to manage relational distance? How far should one be empathetic towards suicidal patients or directive with the setting of care? It was noticed an emotionally context where the tendency of students to identify with the patient was prevalent.

In consequence’s, the groupal dynamic required a great attention on the part of the moderators, to enhancing and building the medical identity of the trainees.

During the second year, in contrast, the emotional climate was quieter, the cases presented in a more balanced way.Trainees were distanced from the patient’s position as victim and accept better identify to the therapists.

**Image 1:**

**Figure:**
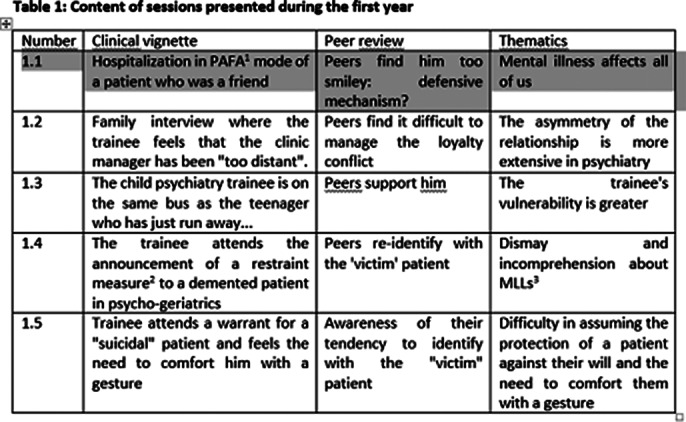

**Image 2:**

**Figure:**
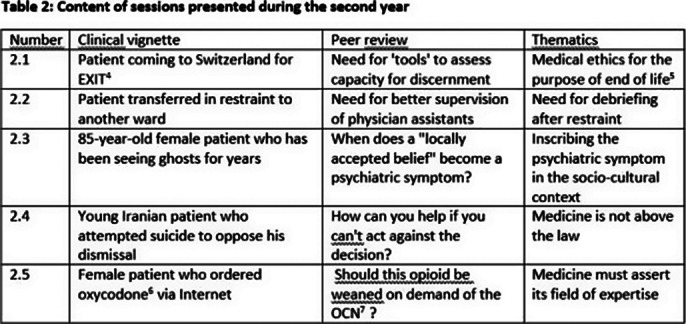

**Conclusions:**

Balint group for medical student during their immersion in the psychiatry field are useful to help students to improve empathy in the patient relationship and to better understanding the specificity of the doctor relationship in psychiatry. But the confrontation with the mental illness and their treatment, especially seclusion treatment, asked a lot of questions from the trainees. The students during the first year of Balint introduction showed a great identification with the patient, essentially catched by the manifest speech and complaints. We observed some changes between the both year, with some hypothesis: better prepared students with previous teaching but also the better frame for their attend by the medical staff in hospital or in ambulatory services.

Further studies must be conducted with qualitative items (satisfaction enquiry by the students, and quantitative findings.

**Disclosure of Interest:**

None Declared

